# A33 EFFECTS OF THE NEXT GENERATION PROBIOTIC, AKKERMANSIA MUCINIPHILA, ON INTESTINAL INFLAMMATION AND BARRIER FUNCTION

**DOI:** 10.1093/jcag/gwac036.033

**Published:** 2023-03-07

**Authors:** J Grondin, H Wang, S Haq, Y H Kwon, L MacNeil, M Surette, W Khan

**Affiliations:** 1 Farncombe Family Digestive Health Research Institute; 2 Department of Pathology and Molecular Medicine, McMaster University, Hamilton, Canada; 3 Beth Israel Deaconess Medical Center, Harvard Medical School, Boston, United States; 4 Department of Biochemistry and Biomedical Sciences; 5 Michael G. DeGroote Institute for Infectious Disease Research; 6 Department of Medicine, McMaster University, Hamilton, Canada

## Abstract

**Background:**

Inflammatory bowel disease (IBD), characterised by chronic intestinal inflammation, is hypothesised to arise from the interplay between susceptibility genes, the immune system, environmental factors, and gut microbiota. *Akkermansia muciniphila* is a symbiotic bacterium that accounts for 1-5% of the human fecal microbiota. This microbe has been hailed as a next-generation probiotic, principally with regard to its plethora of beneficial host interactions, including the ability to influence mucin secretion and strengthen the intestinal barrier.

**Purpose:**

Though a clear-cut role and mechanism by which *A. muciniphila* influences inflammatory conditions is unknown, evidence indicates this microbe is depleted in IBD, suggesting it may have protective effects that are lost in these conditions. Here, we investigate the role and mechanism of *A. muciniphila* in intestinal inflammation and its influence on intestinal barrier function by utilizing barrier-disrupting models of colitis.

**Method:**

Across several experimental models of intestinal inflammation including the chemically-induced dextran sulphate sodium (DSS) model, the parasitic-based model of *Trichuris muris *infection, and the spontaneous *Muc2-/*- model, *A.muciniphila* was administered by oral gavage. Disease activity index, macroscopic scoring and histological scoring were all performed to assess the severity of intestinal inflammation. Various pro- and anti-inflammatory cytokines were assessed within colonic tissue using commercially available ELISA kits.To investigate the effects that *A. muciniphila* has on barrier function in the context of colitis, reverse transcriptase qPCR was used to explore several factors, including several TJPs, AMPs, and mucins. To analyse the composition of the microbiota and changes in diversity with *A. muciniphila* supplementation, 16S rRNA sequencing of fecal samples was performed.

**Result(s):**

Though only minor benefits were derived from this microbe in germ-free mice, in specific pathogen-free (SPF) mice, administration of pasteurized *A. muciniphila* in a DSS recovery model ameliorated inflammation severity and promoted recovery compared to controls. When gavaged prior to DSS administration, both live and pasteurized *A. muciniphila* failed to diminish inflammatory markers indicating minimal preventative effects. *T. muris*-infected SPF mice treated with live *A. muciniphila* showed increased levels of Th2 and anti-inflammatory cytokines, decreased worm burden, and enhanced levels of the mucin, *Muc5ac,* compared with those receiving control broth or pasteurized bacteria. Further, both live and pasteurized *A. muciniphila* ameliorated the severity of inflammation in a mucin 2 deficient (*Muc2*^*-/*-^) mouse model of spontaneous colitis, indicating that these protective effects are Muc2-independent.

**Conclusion(s):**

These observations provide us not only with an enhanced understanding of the role *A. muciniphila* plays in the pathogenesis of intestinal inflammatory conditions but also may fuel novel avenues of treatment for those with IBD.

**Please acknowledge all funding agencies by checking the applicable boxes below:**

CIHR

**Disclosure of Interest:**

None Declared

